# Conceptualisations of mental illness and stigma in Congolese, Arabic-speaking and Mandarin-speaking communities: a qualitative study

**DOI:** 10.1186/s12889-022-14849-4

**Published:** 2022-12-15

**Authors:** Shameran Slewa-Younan, Klimentina Krstanoska-Blazeska, Ilse Blignault, Bingqin Li, Nicola J Reavley, Andre M. N. Renzaho

**Affiliations:** 1 Translational Health Research Institute, School of Medicine, Western Sydney University, Campbelltown, Australia; 2 Centre for Mental Health, Melbourne School of Population and Global Health, University of Melbourne, Melbourne, Australia; 3 Social Policy Research Centre, University of New South Wales, Sydney, Australia

**Keywords:** Culturally and Linguistically Diverse, Mental illness, Mental Health, Stigma, Arabic-speaking, Congolese, Mandarin-speaking, Australia, Qualitative

## Abstract

**Background:**

Australia is an ethnically diverse nation. Research has demonstrated an elevated risk of developing a mental illness in culturally and linguistically diverse (CaLD) communities yet uptake of mental health services is low. To improve mental health treatment seeking and outcomes for CaLD individuals in Australia there is an urgent need to deeply understand barriers to treatment such as stigma. Using an exploratory qualitative approach, the aim of the study was to explore how CaLD communities’ conceptualise and interpret mental illness and associated beliefs and experiences of stigma.

**Methods:**

The study focused on three key CaLD groups: the Congolese, Arabic-speaking and Mandarin-speaking communities residing in Sydney, Australia. A series of eight focus group discussions (*n* = 51) and 26 key informant interviews were undertaken online using Zoom during the period of November to December 2021. Focus group discussions and key informant interviews were digitally recorded, transcribed, and analysed using NVivo software.

**Results:**

Three major themes were identified. The first theme related to mental illness terminology used in the three communities. Despite variation in the terms used to refer to ‘mental illness’ all three communities generally distinguished between ‘mental illness’, a more severe condition and ‘mental health problems’, considered to arise due to stressors. The second theme centred on beliefs about mental illness; with all three communities identifying migration-related stressors as contributing to mental illness. Culturally related beliefs were noted for the Congolese participants with the perception of a link between mental illness and supernatural factors, whereas Mandarin-speaking participants highlighted lack of inter and intrapersonal harmony and failure to adhere to values such as filial piety as contributing to mental illness. The final theme related to mental illness related stigma and the various ways it manifested in the three communities including presence of collectivist public stigma felt across all three groups and affiliate (family) stigma reported by the Arabic and Mandarin-speaking groups.

**Conclusions:**

We found rich diversity in how these communities view and respond to mental illness. Our findings provide some possible insights on both service provision and the mental health system with a view to building effective engagement and pathways to care.

**Supplementary Information:**

The online version contains supplementary material available at 10.1186/s12889-022-14849-4.

## Introduction

Australia is an extremely diverse nation with the most recent 2021 census data indicating just over half (51.5%) of Australians were born overseas (first generation migrant) or had a parent born overseas (second generation migrant; [1]). The term Culturally and Linguistically Diverse (CaLD) is often used to refer to migrants from a non-English speaking background, excluding those from an Anglo-Saxon, Anglo Celtic, Aboriginal or Torres-Strait Islander ancestry [2]. Within Australia, two of the largest CaLD groups are those with Chinese and Arab ancestry and correspondingly, the two most commonly spoken languages in the home other than English in Australia are Mandarin (spoken by 2.7%) followed by Arabic (1.4%; [1]). Australia has multiple migration streams including Skilled pathway, the Family Stream and the Refugee and Humanitarian Program [3]. With respect to refugee and humanitarian entrants, Iraq and Syria have comprised the top source of individuals granted offshore humanitarian visas granted over the past seven years [4]. Another emerging migrant community in Australia comprises individuals from the Democratic Republic of the Congo (DRC), with Australia increasingly resettling those fleeing the worsening civil unrest and violence [5].

Due to greater likelihood of exposure to potentially traumatic events such as torture, murder of family members and friends and other human rights violations, refugee humanitarian entrants are a particularly vulnerable subset of CaLD individuals [6]. They are at significantly greater risk of developing posttraumatic stress disorder (PTSD) and major depressive disorder [7, 8] compared to the general Australian public often as result of increased exposure to trauma events. Post-migration, stressors such as family separation, loss of existing supports, racism, socioeconomic difficulties, and language barriers pose further challenges to the mental health of all migrants irrespective of migration pathways [9–11].

Mental health conditions and substance use disorders were amongst the top five causes of disease burden among Australians in 2018 [12]. There is good evidence to suggest that untreated mental illness and delayed help-seeking results in significant social and economic costs to the individual, health system, and society [13]. Notable disparities exist between how CaLD groups in Australia engage and utilise mental health services compared to non-CaLD, with individuals from the Middle East and Africa demonstrating low uptake of mental health services despite high reported rates of psychological distress [14]. Similarly, despite comparable rates of depression among Chinese and non-Chinese patients in Australia, Chinese immigrants have been shown to under-utilise mental health services compared to non-Chinese individuals [15]. Although CaLD individuals underutilise mental health services in the community, evidence suggest they are disproportionately admitted to mental health units [16]. Relatedly, there is evidence from studies examining longitudinal data on resettled refugees in Australia to suggest that even when professional help is sought the average number of mental health sessions is less than the recommended treatment guidelines [17, 18]. In addition to structural barriers such as financial constraints, transportation difficulties and lack of culturally and linguistically appropriate mental health services [19], differing knowledge and beliefs about mental illness have been suggested to contribute to lower levels of professional help-seeking among CaLD individuals [20]. Researchers have defined knowledge and beliefs about mental illness as well as attitudes toward mental illness as components of ‘mental health literacy’ [21]. Notably, mental illness related stigma has been postulated to play a significant role in CaLD communities engagement with mental health services [19, 22, 23].

Stigma toward people with mental illness is recognised as a significant barrier to seeking help and is associated with low self-esteem and reduced self-efficacy; a “double burden” when experiencing mental illness [24]. Mental illness related stigma is a complex construct and has been extensively researched and elaborated on since Goffman who first categorised it within a social framework as an “attribute that is deeply discrediting” [25]. Generally, the term stigma comprises of multiple components including negative stereotypes (beliefs such as a person with mental illness is dangerous or weak in character), prejudice (emotional reactions such as anger with or fearful of person with mental illness), and discriminatory behaviour towards people with mental illness [26]. For a person living with mental illness, when the public’s negative attitudes are internalised, this can result in self-stigma [26]. Stigma may also be felt by the family of a person with mental illness, in the form of affiliate stigma (internalised stigma felt by family or other associates) or courtesy stigma (public’s stigma towards family/associates of mental ill person) [25, 27, 28]. Research has demonstrated that although stigma is a universal experience, it can manifest differently across cultures and moral contexts [29]. For example, as a result of the collectivistic values that are inherent in African [30, 31], Chinese [32, 33], and Arabic-speaking communities [34] decisions are often made at a family or community level. A sense of self is interconnected with family wellbeing and decisions regarding mental health problems are made by the family/elders not just the individual. In societies that uphold collectivistic values, having a mental illness is a reflection on the person's family and can bring the family shame. For example, affiliate stigma has been noted to be more pronounced in Chinese communities due to cultural values such as ‘face’ and tainted lineage concerns [27, 28]. Other factors that can influence how stigma is manifested across different CaLD groups are culturally related beliefs regarding the aetiology and risk factors for mental illness [35]. For example, the belief that mental illness is caused by demonic or spiritual possession commonly held across different African communities can lead to fearful emotional reactions and place solutions within the purview of traditional or religious leaders [36].

### ‘What matters most’ framework for understanding culture-specific aspects of stigma

This study was informed by ‘What matters most’ framework to understand culture-specific aspects of stigma. Yang and colleagues (2014) postulated a theoretical approach for examining stigma across cultures based on ‘what matters most’ to individuals within a cultural group [27]. They argue that fundamental values that ‘matter most’ in cultures are preserved through everyday activities and social engagements. When an individual member of the group is unable to participate in such activities, it undermines their standing within the local group and, thus, endangers their ‘personhood’. This theory emerged from Yang et al. (2014) when they interviewed 50 Chinese immigrants living in New York City, USA with a lived experience of mental illness. In this study, it was noted that mental illness stigma was related to the degree to which they were able to participate in work given that the accumulation of financial resources in that specific cultural context was ‘what mattered most’ [27]. Consequently, the Yang argues that ‘What matters most’ framework can assist in identifying culturally specific dimensions of stigma by exploring the interactions between cultural norms, roles, and values that impact personhood.

### Current study

There is limited research into conceptualisations of mental illness and associated beliefs and experiences of stigma, in the Congolese, Arabic-speaking and Mandarin-speaking communities in Australia, and to our knowledge, there is no study to date comparing the views of people in these three communities. Given that Mandarin and Arabic are the two most spoken languages in Australia other than English, and the emerging Congolese community, such an investigation is warranted to inform targeted stigma reduction initiatives and other mental health service provision for these specific CaLD communities.

## Methods

### Design and study context

This exploratory qualitative research was couched within a larger study investigating views on mental health help-seeking including that of stigma. All research was performed in accordance with relevant guidelines and regulations of Western Sydney University Human Research Ethics Committee (approval number H14608). All researchers had qualifications in health, or a health‐related discipline obtained in their country of origin, Australia or in both countries.

Six bilingual health workers from the target communities were employed and trained in the recruitment, screening, and interviewing processes. Triangulation of data sources (community members and community leaders) and methods (qualitative individual interviews and focus groups for each community group) contributed to rigour and enabled the researchers to explore a broad range of perspectives and make comparisons within and between the three CaLD communities. The COREQ 32 [37] consolidated criteria for qualitative research (Appendix A Table 1A) is utilised to report the current study.

### Participants

Recruitment was conducted using a combination of purposive and snowball sampling [38]. Participants were recruited using the networks and contacts of the relevant investigators (AR, BL, SSY) in addition to those of the bilingual health workers. Focus groups were drawn from existing networks and included males and females. In relation to recruitment for key informant interviews, these participants were identified through their involvement with the community via relevant community-based organisations. Following the dissemination of translated flyers on the study and via networks, interested participants were instructed to contact the bilingual health workers. Then, bilingual health workers conducted screening procedures and preparation phone calls with interested potential participants. A relationship was established with participants prior to study commencement during the screening procedure and preparation phone calls. Participants were informed of reasons for doing the research at screening and at the beginning of the focus group discussions/informant interviews.

Participants were eligible to be part of focus group discussions if they were Arabic speaking, Mandarin-speaking or Congolese individuals with lived experience of mental illness including those of carers, or family members. All participants were 18 years of age or older, residing in Sydney and had arrived in Australia no more than seven years ago. However, some of the Mandarin-speaking community participants had arrived in Australia earlier than seven years but due to back-and-forth travel to China had not spent longer than a total of seven years residing in Australia.

Participants were eligible to participate in the key informant interviews if they were identified as a leader within the three groups and had informed perspectives regarding their communities. This included both formal or informal roles with examples being community elders, religious leaders or holding role as community worker within an ethnic specific and/or trusted organisation. These participants were also required to be 18 years of age or older and residing in Sydney.

### Data collection

A series of 8 focus group discussions (*n* = 51) and 26 key informant interviews with Arabic-speaking, Mandarin-speaking (Chinese) and Congolese community members and leaders residing in Sydney were conducted during November and December 2021. The bilingual health workers conducted the interviews or focus group discussions. Due to the COVID-19 Delta outbreak in Sydney and restrictions, all focus group discussions and key informant interviews were conducted remotely using Zoom and digitally recorded. Each focus group discussion was facilitated by two bilingual health workers. The facilitator was not only central to the discussion and its moderation, but they also created a relaxed and conformable Zoom environment to better manage relationships between unfamiliar participants. The second person’s role was to take notes in order to complement the audio files, managed the Zoom meeting by ensuring everyone participated, and to observe non-verbal interactions and group dynamics. Prior to the focus group discussion, sociodemographic data of consented participants was collected via a preliminary phone call by a bilingual health worker, during which they also ensured the participant has access to Zoom and understood how to operate it. The focus group discussions lasted approximately 90 min and at the end of each main section of the discussion, the bilingual health worker summarised the content to ensure the participants’ perspectives were obtained and interpreted correctly, a verification process that enhanced the credibility of the findings. The key informant interviews lasted between 20 to 40 min and were undertaken by a bilingual health worker using the interview guide. The interview guides included open ended questions formulated using a review of literature and input from the primary researchers. The interview guides which formed part of the larger qualitative research project focused on generating an understanding of each community’s conceptualisation of mental illness, including beliefs about aetiology and risk, barriers to seeking treatment such as stigma and mental health help-seeking treatment preferences. These interview guides included open ended questions formulated using a review of literature and input from the primary researchers, an approach successful used in past [39]. For the purposes of this current study, responses regarding conceptualisation of mental illness and mental illness related stigma are reported upon. All focus group discussion participants received a $30.00 electronic supermarket voucher as reimbursement for their time in participating in the research.

### Data analysis

Interviews and focus group discussions were transcribed into English by the bilingual health workers for data analysis. The bilingual health workers listened to the audio recordings and subsequently translated the interviews from the original language to English, with the added task of checking and re-checking the translations as required. Thematic analysis, using NVivo 12 software, was conducted in accordance with published guidelines for interpretative phenomenological analysis (IPA) [40] in order to understand the lived experiences of study participants and their views on mental illness and help-seeking and barriers including mental illness related stigma. IPA takes a phenomenological and interpretative approach in which it seeks to understand the subjective account of the phenomenon of those researched, but always recognising the centrality of the researcher’s own experiences and theoretical positioning in making sense of that account [41]. Analysis was conducted systematically in that the data from the Congolese group were analysed first, then the Arabic-speaking group, then the Mandarin-speaking group. Analysis was undertaken by KKB, SSY and an experienced research officer and bilingual health worker (YL). Regular weekly meetings were held to consider aspects of analysis including that of reflexivity, also addressed through note keeping. The bilingual health workers and AR were consulted continuously through the process to clarify transcriptions and words. This process was not linear but moved back and forth between members of the team who transcribed (bilingual health workers) and those who were conducting analysis. Coding was conducted separately by YL and KKB with consistency and discrepancies resolved at weekly supervision meetings with SSY. Inter-rater agreement was assessed using quadratic weighted Gwet’s AC1 [42]. Gwet’s AC1 and percentage of agreement were 0.88 (95%CI: 0.56, 1.00) and 0.89 (95%CI: 0.63, 1.00) respectively, suggesting excellent agreement. Initial codes were then grouped into categories. The fourth stage was to search for connections between categories thus generating themes and subthemes. This process was then repeated for the next transcript. Once analysis had been completed for each transcript, a final master list of themes was generated. At this final stage, a thematic analysis across the interview types and the community groups was undertaken to generated the final results. In line with the broad principles for IPA rigour, findings were presented by SSY and KKB at regular meetings with the primary researchers (IB, AR, BL and NR) as a form of peer critique where descriptive validity and transparency of interpretation was sought [41].

## Results

The socio-demographic characteristics of the participants are presented in Table 1. A total of 77 individuals participated in the study. Three Arabic-speaking focus group discussions with 6 participants in each were conducted. Three Mandarin-speaking focus group discussions, with 6 participants, 7 participants, and 8 participants, respectively, were conducted. The majority of focus group discussion participants in the Arabic-speaking and Mandarin-speaking communities were female and fell within the 18 to ≥ 50 age range. Two Congolese focus group discussions with 4 participants and 8 participants, respectively, were conducted. Most focus group discussion participants in the Congolese community were male and most fell within the 30 years or younger age range. With regard to key informant interviews, all participants were aged 30 years or older.

**Table 1:** Sociodemographic characteristics of participants

	**Arabic-speaking Community**^**#**^		**Mandarin-speaking Community**^**^**^		**Congolese Community**^**a**^	
	Focus group discussions	Key informant Interviews	Focus group discussions	Key informant Interviews	Focus group discussions	Key informant Interviews
**Characteristics**	*n*	*n*	*n*	*n*	*n*	*n*
Gender						
Male	5	7	6	2	8	4
Female	13	3	15	6	4	4
Age Group						
18-30	3	-	1	-	9	-
30-39	4	-	6	2	2	-
40-49	7	2	3	3	-	4
≥50	4	8	11	3	1	4
Country of Origin						
Middle East^b^	18	9	-	-	-	-
Australia	-	1	-	-	-	-
China	-	-	21	8	-	-
Sub-Saharan Africa^c^	-	-	-	-	12	8
Years in Australia						
<5	14	-	6	-	7	-
≥5	4	-	15	-	5	-

^#^ YM and TH facilitated the Arabic-speaking focus group discussions, YM facilitated the Arabic-speaking key informant interviews

^^^ YL and JL facilitated Mandarin-speaking focus group discussions, YL facilitated the Mandarin-speaking key informant interviews

^a^AR and VM facilitated the Congolese community focus group discussions, NZ facilitated the Congolese community key informant interviews

^b^ Iraq, Jordan, Egypt, Lebanon, Syria

^c^DRC, Rwanda, Nigeria

### Theme 1: mental illness terminology

There was considerable variation in mental illness related terminology used across all three communities in both the focus group discussions and key informant interviews. Participants utilised various terms to refer to ‘mental illness’ and used these interchangeably with no consistency. However, it was clear that all three communities generally distinguished between ‘mental illness’, a more severe condition and ‘mental health problems’ or ‘mental issues’, commonly considered to arise due to stressors.

*“They are afraid that a mental health problem is a mental illness. In this case, Mandarin-speaking people try to protect themselves from others and try not to let other people know they have a mental illness.”* (Mandarin FGD2, Female 60)

This distinction in the Arabic-speaking participants is important to note because there was a strong belief that mental illness was a hopeless, chronic and an incurable condition. One focus participant stated:

*“In our society, the mentally ill is considered a hopeless condition and will never be treated or recovered. They will not accept it as a common disease, make it a shameful situation.”* (Arabic FGD1, Female, 35)

By holding such views, individuals are less likely to see value in seeking help leading to possibility of a worsening in outcomes and prognosis over time and thus reinforcing perceptions of incurability.

*“We do not think the mentally ill person will recover. But we trust that this person once mentally ill, will be forever ill!”* (Arabic FGD2, Female, 47)

An interesting tension emerged in the views of the Congolese community with respect to mental illness. Both community leaders and focus group participants discussed the widespread traditional belief that mental illness is taboo and as such does not exist. and yet symptoms of mental illness were frequently described.

*“There is this state of suspicion in my community that people first of all don't recognise that mental health [illness] exists.”* (Congolese Interview, Health Care Worker Male 44)

However, symptoms of mental illness, thought to be observable and usually not concealable to others were frequently described.

“*They [people with mental illness] act different from normal people*” (Congolese FGD1, Female, 19).

Overall this variation in terminology when discussing mental illness provides valuable insight into preferred expressions of mental distress. In particular, it is clear that there is a strong attachment of stigma to the label of ‘mental illness’ in all three communities. While greater contextualisation is required to identified preferred terms when working within communities, it would seem that ‘mental health problems’ was more digestible to participants.

### Theme 2: cultural health beliefs

A notion that mental illness manifests when stress exceeds coping ability was discussed by all three communities, with resettlement challenges such as unemployment, loss of support structures, language barriers and so on consistently mentioned as either contributing to the development or worsening of mental illness.

*“The experience in Australia, for instance, someone who's driving and get a fine, they don't know how to pay, or they lose their driving license, develop mental health because you lose employment, you don't know how to cope, all those things are associated to mental health.”* (Congolese Interview, Community Leader, Male, 50)

*“Our supports are very limited. Not only do we need to support our children to go out and come back, but we also need to do chores and our work and everything for our career. We could easily get frustrated. But, then, what does it look like in China? There are many other supporting hands under the base hands, including relatives, parents, parents-in-law, and grandparents.”* (Mandarin Interview, Mental Health Worker, Female, 30+)

*“We are new here in Australia, and we get annoyed because we don’t know the language and people make fun of you because of it.”* (Arabic FGD3, Female, 53)

Changes to the traditional family structure and distinct gender roles, which were clearly differentiated in Africa, following migration were described by some Congolese focus group participants as contributing to lowered self-esteem and loss of identity for males. As a result, a sentiment that mental illness disproportionally affect males more compared to females was expressed.

*“When you get here in Australia, you have this thing where, I don’t think it necessary a bad thing women too can go and work. They can also go and do these things. This is well and good but then the fact that women can do these stuffs, the role of a men start to get degraded” (Congolese FGD2, Male, 21)*

In addition to stressors associated with resettlement, the Congolese and Arabic-speaking participants discussed the additional unique challenges associated with being from a refugee and asylum-seeker background with the role of trauma featuring strongly as a contributing to mental illness.

*“Congolese migrated to Australia, most of them came through offshore humanitarian settlement, they are refugees, they are already traumatized. And you can see that based on the trauma that they had in the refugee camp, there was not much support. So they carry the trauma and when they arrive to Australia, in a new country, they’re again traumatized by moving from Congo to the refugee camp, from the refugee camp to Australia, so there is that shock and trauma.”* (Congolese Interview, Community Leader, Male, 50)

The impact of prolong trauma was also highlighted.

*“I just want to point out that all the communities are affiliated, like the Iraqi communities, whether they are Muslims, Assyrian, Chaldean, Mandean etc, they live in the same community, the same shell, and almost have the same mental health status. All of such happens as a result of wars consequences, forced displacement effects, the effects of mental stress, and as a result of everything that happened before 2003 and after 2003. These are all accumulations of the difficult conditions that have been experienced by this region and its people.”* (Arabic Interview, Community Worker & Religious Leader, Male, 59)

Beyond migration related stressors, the Congolese and Mandarin-speaking communities discussed the role of other factors in developing mental illness that had more culturally specific origins. Both focus group participants and leaders in the Congolese community acknowledged that when an individual is experiencing mental illness, some people may attribute it to supernatural causes. Words such as ‘mapepo’ (Swahili for demonic possession), witchcraft, and poison were used to refer to supernatural causes. The participants highlighted that such a belief is commonplace not only amongst some Pastors, but also family members and friends.

*“Because when somebody is experiencing something like that, it means the family would start to think it witchcraft rather than thinking it mental illness. Which is a big problem.”* (Congolese FGD1, Male, 59)

By contrast, supernatural or spiritual causes were rarely described in the Arabic-speaking focus group discussions, which is inconsistent with previous research that has demonstrated its prevalence in Arabic-speaking society [10]. However, disengagement from religion or absence of spiritual comfort was highlighted as contributing to the development or exacerbation of mental illness by some Arabic- speaking leaders.

*“Whenever this person is distant from his spiritual and religion, the more he will suffer from mental problems”* (Arabic Interview, Community Leader, Male, 56)

The importance of inter and intra personal harmony and disruptions to such were highlighted as causing mental illness amongst the Mandarin-speaking participants. Disharmony in the family unit, and in particular the parent–child relationship, was linked to cultural tensions that may arise due to cultural and generational differences. For example, how a child for example adheres to values such as filial piety. Participants noted that seniors especially will attribute their emotional distress to their children not adhering to cultural values rather than labelling their emotional distress as mental illness.

*“I once have met a family, and I am very close to that family. The parents have always told me that their children were unfilial and asked them to do many things. Sometimes, they quarrel with each other badly…For young people, " If I have a bad temper, I may have mental health problems". The young people are willing to see a psychologist, but for the seniors, they won't. The seniors always say, "I am this kind of person. My children are supposed to be kind to me. They are supposed to do this and that". They blame a lot of the problems on their children and refuse to admit what problems they have. Maybe they are irritable, but they just won't admit that they may have mental health issues.”* (Mandarin Interview, Community & Organisation Leader, Male, 70+)

Other more culture-specific beliefs about mental illness were offered again emphasising the importance of harmony, this time within the individual.

*“In Mandarin-speaking people's concept, they always think I may be off-balance. They use 'off balance' to explain many symptoms, whether it's physical or psychological.”* (Mandarin Interview, Mental Health Worker, Female, 30+)

Other attributes applied to those with mental illness seemingly suggested unpredictability and personal responsibility.

*“They want to do one thing, but they will change their mind in a minute. Or they’ve made a decision, but then they will overturn their decision quickly. This decision must be wrong. There are some new problems. In short, they blow hot and cold.”* (Mandarin FGD1, Female, 60+)

### Theme 3: stigma and its variations

The issue of stigma was commonly raised by participants in all three CaLD groups as a significant barrier to help-seeking. Common to all three communities was that openly speaking about or identifying mental illness was considered ‘taboo’.

*“Our misunderstanding that mental health illness is a taboo topic or a secretive issue, no one should know about it or talk to anyone about it because we still consider mental health or psychological issues are madness.”* (Arabic Interview, Community Leader, Male, 68)

*“You are born you grow up you never hear about a certain something [mental illness], they start telling you about that thing you feel like “no,” they feel like it is something like kind of taboo*.*”* (Congolese Interview, Community Leader, Female 41)

*“In addition, it is not easy for people to speak up about their problems. It’s like a taboo for Chinese, right?”* (Mandarin FGD2, Female, 69)

However, other more specific manifestation of stigma seemed to be influenced not only by the communities conceptualisation of mental illness but also by their traditional values. In this way the ‘what matters most’ theoretical framework [27] is useful to operationalise these more culture-specific aspects of stigma and their contextual environment effect.

The importance of family reputation and honour in the Arabic-speaking community is strongly upheld [43]. Consequently, mental illness is seen as leaving a ‘mark’ on one’s identity as well as their family’s identity and thus bringing dishonour and ‘Aár’ (Arabic word for shame related to mental illness)—to the individual and family. Consequently, participants reported families will hide the individual with mental illness or presence of mental illness in the family from the community to maintain reputation and status.

*“His family tries to hide it from the community, they are aware of any rumours that could spread because of this and the reputation will be that this person in the family is mentally ill.”* (Arabic FGD2, Female, 34)

Participants alluded to the strong presence of affiliate stigma in the Arabic-speaking community, that is, the extension of stigma to the family members of the person with mental illness, further motivating need for secrecy.

*“The mentally ill person daughters or sisters will lose the opportunity in getting married even his relatives will lose a good chance in getting married”* (Arabic Interview, Community Leader, Male, 56)

A view that disclosing mental illness is associated with losing ‘face’, and that such a ‘domestic shame’ should not be made public was reported by the Mandarin-speaking focus group participants and leaders.

*“Maybe they are afraid of losing face. The traditional mindset is that domestic shame should not be made public.”* (Mandarin FGD1, Female, 39)

Relatedly, was the belief that mental illness is associated with a loss of occupational or social functioning and therefore threatens social capital and reputation. Therefore, mental illness is often denied because it is associated with losing ‘face’ and reputation in the community.

*“One important thing is that if someone has been diagnosed with mental illness, they will not tell us, and their children will not tell us either, as many Mandarin-speaking people care about their ‘face.’”* (Mandarin Interview, Community Organisation Leader, Male, 70+)

Due to the general belief that religion and community leaders play an important role in addressing mental illness in the Congolese community, the importance of trust was highlighted. Some focus group participants who expressed a concern that when mental illness is addressed at this wider network level it may lead to gossip and invasion of privacy.

*“You could be depressed but you will be wondering by yourself who can I talk to, who can I trust? To tell how I am feeling? You know, you are scared?”* (Congolese FGD1, Female, 19)

Thus self-isolation and withdrawal was seen as a possible response to evade stigmatisation.

*“That is why is so hard to identify people who are mentally ill in the Congolese community, because you won’t know if this person is staying home because they are depressed or because they’re running away from gossip.”* (Congolese FGD1, Female, 19)

This strong adherence to religion to alleviate mental illness, was also seen by some of the younger focus group participants as minimising and dismissive.

*“I think people come to you and tell you these things [mental health issues] straight out they don’t they don’t really hide it but I think – this might sound bad I like to be a rational person usually – I think that religion yeah kind of cloud our judgement. How does it cloud our judgement? I come to you and tell you aunty life, life is difficult I am going through all these stuffs and at school I am not doing well. You know our child, God is there go there and there. You know they start making it about prayers...we use religion to dismiss it.”* (Congolese FGD2, Male, 21)

Finally, pride and the failure to espouse the traditional roles of African men as being the provider and ‘a strong person’ was highlighted by community leaders as having a significant impact on men not disclosing their mental illness.

*“Like men, for example, with men feel proud of themselves rather than coming up and say I'm suffering from this, they would rather keep quiet, and the more they keep quiet, that particular mental health [illness] keeps eating them up.”* (Congolese Interview, Health Care Worker, Male 44)

## Discussion

Previous research has demonstrated that stigma towards people with mental illness is common in CaLD communities in Australia and this is supported by the findings of the current study in relation to the Arabic-speaking, Mandarin-speaking, and Congolese communities. Our study provided further insights into the conceptualisations of mental illness and how such conceptualisations may be related to stigma. Notably, we found variations in terminology across all three communities when referring to mental illness. However, in spite of this variation of terms used by participants, it was clear that all three communities generally distinguished between ‘mental illness’ and ‘mental health problems’, with the former denoting more severe conditions and the latter term denoting more common conditions such as anxiety and depression, a finding not dissimilar to that noted in the general Australian public [44]. We also found that migration-related stressors were highlighted by all three communities as playing an important role in contributing if not causing mental illness. This is consistent with previous research where factors such as separation from family, limited English proficiency and experiences of racism can be associated with increased psychological distress and vulnerability to mental illness [9–11]. As such stigma reduction initiatives for CaLD communities should be cognisant of the language and terminology used, with our findings suggesting community members found a focus on specific symptoms such as “stress” more digestible than diagnostic labels as it related to their universal experiences of migration. However, further research is needed to understand the implications of incorporating a more psychosocial explanation on measures of stigma. This is especially important as evidence is increasingly demonstrating the unintended outcome from public health campaigns in the general public where biomedical models to explain mental illness in an effort to reduce blame can lead to increase aversion and perceptions of dangerousness [45].

Overall, we found support for the role of traditional beliefs regarding the factors thought to contribute or exacerbate mental illness, which was in line with previous research. Similar to the current findings, Chinese migrants living in Melbourne pointed to psychosocial problems such as life stress and interpersonal conflict as causes of depression [46]. An emphasis on the role of being ‘off balance’, in other words, having an imbalance of ‘qi’ (Chinese term for vital energy) as contributing to mental illness was also noted in the Mandarin-speaking community findings, consistent with traditional Chinese medical view of mental illness [47]. Within the Congolese participants, the influence of supernatural beliefs such as demonic possession and witchcraft were noted, consistent with previous research where such factors were reported to cause mental illness [48]. Such beliefs were endorsed to a lesser extent by younger participants, who expressed a desire for a broader perspective of mental health, which may point to the role of acculturation in changing views. However, the finding that the Arabic-speaking community members did not report supernatural or religious causes of mental illness, is inconsistent with previous research which has found that religious and supernatural attributions of mental illness were common among Arabic-speaking individuals and related to higher levels of stigma [43, 49]. While religion is well recognised as playing a significant role in Arabic-speaking communities, individuals differ in levels of religiosity and this may account for our unexpected finding. Consequently, our findings suggest that while cultural beliefs play an important role in how CaLD communities perceive and conceptualisation mental illness, the rich diversity of these communities is evident and should not be neglected when working in this space.

In relation to the role of stigma in the three communities, again similarities and differences emerged. From the Congolese interviews and focus group discussions, public stigma and internalised stigma was strongly evident, with family stigma less so. A systematic review by Misra and colleagues [50] found that among 18 studies involving Black Americans, internalised and public stigma were more commonly reported compared to family stigma. Furthermore, we found a culture-specific aspect of stigma related to religion and the role it played in care. In line with Yang and colleagues [27] research summarising culture-specific aspects of stigma in African American groups, the authors note that the value placed on strong ties to faith and self-reliance contributes to the belief that mental illness can be addressed by religious institutions and church-based coping or prayer [27, 51]. Such behaviour may also be related to the structural inequities in health care faced by African individuals and low faith in the cultural competency and helpfulness of Western mental health services. A sense of disillusionment with the Australian system and loss of traditional roles may also hinder engagement with the broader Australian health system. Recent research into the barriers to mental health help-seeking among African migrants in South Australia also found there was a lack of perceived benefits of mental health care which was perceived as culturally incongruent [48, 52]. Furthermore, the construct of gender was found to be related to culture-specific aspects of stigma. Challenges to the traditional family structure and the distinct gendered role of being the breadwinner posed as threats to ‘personhood’ and were acutely felt aspects of stigma for some males.

Within the Arabic-speaking community public, family, and internalised stigma were noted. Importantly, there was a belief that mental illness as a condition is associated with hopelessness and incurability. Sayed [53] noted there is an implicit assumption in the expectations of Arab patients when it pertains to therapeutic doctor/patient relationships, leading to a sense of passivity towards treatment and the omniscient perception of the doctor to “cure” one’s illness, which is at odds with the collaborative process which defines much of the Western psychotherapeutic approaches and invites the client to be an active agent in their healing. The cultural association that mental illness is incurable and hopeless poses questions about the meaning and concept of recovery or healing in the Arabic-speaking community as well as the expectations held towards professional help-seeking sources. The influence of both pre-migration and post-migration experiences that may influence concept of healing and or recovery require further investigation. The role of family (affiliate) stigma played an important part in motivating the need for secrecy because the value of individual, and by extension, the family’s reputation in Arab culture is impacted by mental illness. There is some suggestion that such secrecy may provide a protective function and buffer against the negative consequences that may arise in case the individual self-discloses information which can be shameful and damaging to the moral status of the individual and family unit and threatens sense of belonging in the community. Future research may investigate the association between authoritarian attitudes and actions toward family members with mental illness and independent/interdependent self-construal among Arabic-speaking individuals.

Types of public, internalised and family stigma were also reported by the Mandarin-speaking participants. In terms of public stigma, specifically stereotypes and beliefs about mental illness such as notions of personal responsibility were evident as well as a component of unpredictability of mental illness. Thus the highly regarded cultural values of self-reliance and restraint means that deviation from such is viewed as a “symbolic threat to societal behaviour” [54]. Similarly, the value placed on regulating one’s behaviour to maintain harmony was also evident in aspects of stigma in the Mandarin-speaking participants. The concept of filial piety, and deviation from, played a role in how Mandarin-speaking seniors viewed mental illness. Another culture-specific aspect of stigma related to the Chinese community was the concept of ‘face’ and the relationship between mental illness and loss of reputation, a finding that has been previously reported [27].

The current study’s findings have some implications for the creation and implementation of stigma reduction initiatives in these three communities. There is strong evidence to suggest that education campaigns that incorporate contact with people with lived experience of mental illness can help correct myths and misconceptions as well as dispel stereotypes associated with mental illness that impede help-seeking [55]. However, such campaigns require targeted education accompanied with well supported contact opportunities developed to address the specific beliefs and associated manifestations of stigma in the communities. For example, the belief that mental illness is usually not concealable indicates there may be lack of knowledge that concealable mental illness, typically mild or moderate mental illness that does not impede functioning, exists within the Congolese community. Furthermore, there may be minimal opportunities to learn that most common mental illness is concealable. This is because individuals who choose to disclose their concealable mental illness may be dismissed, leading to less contact opportunities with such individuals, and further reinforcing the stereotype that mental illness is usually observable and extreme. Increasing contact with persons with mild and concealable mental illness may help to disconfirm such culturally specific stereotypes [50]. Other factors which need to be considered is the role of religion and how some participants perceived responses such as prayer and spiritual guidance as being dismissive of experiences of mental illness. These are all complex factors that require further research as they will greatly impact the creation and effectiveness of stigma reduction interventions for the Congolese community in Australia. The belief expressed by the Arabic-speaking participants that mental illness is hopelessness and incurable can be explicitly addressed by increasing contact with individuals who can challenge the stereotype of what recovery and treatment can look like for someone with mental illness and thus contribute to positive attitudinal changes [56].

Addressing the challenges of mental illness stigma in CaLD communities requires systems approach with clinicians and community organisations playing important role in reducing stigma. For example, service providers should be mindful of how they utilise and apply psychiatric labels when working with individuals from these three communities. The provision of a psychoeducation session which takes a Western biomedical approach with a focus on psychiatric labels and biochemical causal model may not be salient to their views or may further risk stigmatising individuals with mental illness when considered in the context of their cultural beliefs and experiences. The finding that participants were reluctant to apply the label ‘mental illness’ when describing mental illness and their personal experiences also needs to be considered when creating stigma reduction initiatives. While avoiding the label mental illness to encourage uptake and acceptability of stigma reduction interventions carries the risk of further perpetuating stigma, such an approach may need to be employed in the initial stages of stigma reduction initiatives until attitudes start to shift and stereotypes of mental illness (e.g., mental illness is a hopeless and incurable condition) are challenged.

### Limitations and strengths of the study

There are several limitations to note when interpreting the current findings. Although we used a triangulation approach to explore broad perspectives, the sociodemographic characteristics of participants should guide interpretation of the findings and impact the generalisability of the results. Participants resided in metropolitan Sydney and the generalisability of our results to community members residing outside the Sydney region are limited, in addition to individuals residing in Australia for longer periods of time. Although recruitment was enhanced through a combination of purposive and snowball sampling using the networks and contacts of the investigators and bilingual health workers, the possibility remains that individuals who are more active members in the community such as those who attend Church groups were recruited as opposed to members less active in community networks. Participants voluntarily participated in the research knowing it was investigating mental health and stigma. This may mean the subset of people we included in the study are individuals who hold more open attitudes toward mental health and illness and a desire to learn more about mental health or illness or have had more contact with people with mental illness compared to individuals in the community who may not have volunteered. Furthermore, there is a possibility of social desirability bias in the results given key informant interviews were conducted with community and religious leaders whose views are well regarded in their respective community. In terms of methodological limitations, participants did not have the opportunity to review and check transcripts due to time and funding constraints, however interviewers did summarise the content of what was discussed at the end of each of the main sections of the interviews to ensure the participants’ perspectives were obtained.

Notwithstanding these limitations, the current study had several strengths. Firstly, the research team and chief investigators were well integrated in the respective communities allowing trust. Secondly, the interviews and focus group discussions were held in the respective language for each community which allowed for the inclusion of newly-arrived individuals who may have otherwise been excluded due to low English proficiency. The use of online technology (Zoom) whilst in the midst of a prolonged COVID-19 related lockdown is another strength and goes towards demonstrating innovation and adaptability of both the research team and participants [57]. Finally, in undertaking community consultations by training bilingual health workers, we were able to build capacity within the CaLD groups and foster advocacy skills.

## Conclusions

Our findings provide crucial insights into how Arabic-speaking, Mandarin- speaking and Congolese communities in Australia view mental illness, including beliefs on risk factors and the role such views have on stigma. Across all three groups, we found migration-related stressors both pre and post arrival to Australia, were commonly reported as contributing to mental illness, while the influence of culturally related health beliefs featured more prominently in the Mandarin-speaking and Congolese groups. Finally, stigma was a commonly reported in the three communities although the manifestation and consequences of stigma differed. Understanding the richly diverse nuances in how CaLD communities view and respond to mental illness is the necessary first step, service providers and mental health systems require in order to build effective engagement and pathways to care. Future research using large scale survey methods are required to examine the extent to which these findings apply to target communities elsewhere in Australia including regional locations.

## Supplementary Information

**Additional file 1:** **Appendix A. Table A1.**

**Additional file 2. **Interview Guides.

## Data Availability

The data sets are not publicly available as they contain information that could potentially re-identify individuals but are available from SSY upon reasonable request and with relevant ethical approval.

## Abbreviation

CaLD: Culturally and linguistically diverse
