# Supplementary material for: Conceptualisations of mental illness and stigma in Congolese, Arabic-speaking and Mandarin-speaking communities: a qualitative study
Source: BMC Public Health. 2022 Dec 15;22:2353. doi: 10.1186/s12889-022-14849-4 (PMC9753024; doi:10.1186/s12889-022-14849-4)
Supplement: Supplementary file 1 — Additional file 1: Appendix A. Table A1. [file 12889_2022_14849_MOESM1_ESM.docx]

### **Appendix A. Table A1.** Consolidated Criteria for Reporting Qualitative Studies (COREQ): 32-Item Checklist.

| Domain 1: Research team and reflexivity | |
| --- | --- |
| Personal characteristics | |
| 1. Interviewer/Facilitator | **Which author/s conducted the interview or focus group?**  YM, TH, AR, VM, YL, JY, NZ |
| 1. Credentials | **What were the researcher’s credentials?**  All researchers had qualifications in health, or a health‐related discipline obtained in their country of origin, Australia or in both countries. |
| 1. Occupation | **What was their occupation at the time of the study?**  Associate Professor (SSY; IB)  Distinguished Professor (AR)  Professor (BL; NL)  Research Assistants (KKB; YL; JY; NZ; YM; VM; TH) |
| 1. Gender | **Was the researcher male or female?**  Female (SSY; KKB; IB; NC; BL; TH; VM; YL; JY)  Male (AR; YM; NZ) |
| 1. Experience and training | **What experience or training did the researcher have?**  Researchers were experienced in conducting qualitative research. SSY and KKB provided training to bilingual health workers. |
| Relationship with participants | |
| 1. Relationship established | **Was a relationship established prior to study commencement?** Yes, during the screening procedure and subsequent phone calls. |
| 1. Participant knowledge of the interviewer | **What did the participants know about the researcher? e.g. personal goals, reasons for doing the research**  Participants were informed of reasons for doing the research at screening and at the beginning of the focus group discussions/informant interviews. |
| 1. Interviewer characteristics | **What characteristics were reported about the interviewer/facilitator?**  See participants and procedure section. |
| Domain 2: study design | |
| Theoretical framework | |
| 9. Methodological orientation and Theory | **What methodological orientation was stated to underpin the study?**  An interpretative phenomenological analysis (IPA) approach |
| Participant selection | |
| 1. Sampling | **How were participants selected? e.g. purposive, convenience, consecutive, snowball**  A combination of purposive and snowball sampling |
| 1. Method of approach | **How were participants approached? e.g. face-to-face, telephone, mail, email**  Telephone and email |
| 1. Sample size | **How many participants were in the study?** 77 |
| 1. Non-participation | **How many people refused to participate or dropped out? Reasons?**  One participant completed the screening procedure and preparation phone calls but did not attend the scheduled focus group discussion |
| Setting | |
| 1. Setting of data collection | **Where was the data collected? e.g. home, clinic, workplace**  Workplace and home as interviews and focus group discussions were conducted online via Zoom due to the COVID19 outbreak |
| 1. Presence of non-participants | **Was anyone else present besides the participants and researchers?**  No |
| 1. Description of sample | **What are the important characteristics of the sample? e.g. demographic data, date**  Sociodemographic characteristics presented in Table 1 |
| Data collection | |
| 1. Interview guide | **Were questions, prompts, guides provided by the authors? Was it pilot tested?**  Yes |
| 1. Repeat interviews | **Were repeat interviews carried out? If yes, how many?**  No repeat interviews were carried out |
| 1. Audio/visual recording | **Did the research use audio or visual recording to collect the data?**  Audio recording was collected |
| 1. Field notes | **Were field notes made during and/or after the interview or focus group?**  During and after |
| 1. Duration | **What was the duration of the interviews or focus group?**  The focus group discussions lasted approximately 90 minutes and the key informant interviews lasted between 20 to 40 minutes. |
| 1. Data saturation | **Was data saturation discussed?**  No – *as this was not part of the data collection approach used* |
| 1. Transcripts returned | **Were transcripts returned to participants for comment and/or correction?**  No |
| Domain 3: Analysis and findings | |
| Data analysis | |
| 1. Number of data coders | **How many data coders coded the data?**  Three – KKB, SSY, YL. |
| 1. Description of coding tree | **Did authors provide a description of the coding tree?**  Yes |
| 1. Derivation of themes | **Were themes identified in advance or derived from the data?**  Derived from the data as well as the theoretical knowledge and practical experience of the data analysts |
| 1. Software | **What software, if applicable, was used to manage the data?**  NVivo 12 Software for Windows |
| 1. Participant checking | **Did participants provide feedback on the findings?**  No |
| Reporting | |
| 1. Quotations presented | **Were participant quotations presented to illustrate the themes / findings? Was each quotation identified? e.g. participant number**  Yes – see results section |
| 1. Data and findings consistent | **Was there consistency between the data presented and the findings?**  Yes – see results section |
| 1. Clarity of major themes | **Were major themes clearly presented in the findings?**  Yes – see results section |
| 1. Clarity of minor themes | **Is there a description of diverse cases or discussion of minor themes?**  Yes – see results section |
