# Supplementary material for: Conceptualisations of mental illness and stigma in Congolese, Arabic-speaking and Mandarin-speaking communities: a qualitative study
Source: BMC Public Health. 2022 Dec 15;22:2353. doi: 10.1186/s12889-022-14849-4 (PMC9753024; doi:10.1186/s12889-022-14849-4)
Supplement: Supplementary file 2 — Additional file 2. Interview Guides. [file 12889_2022_14849_MOESM2_ESM.docx]

Interview Guides

Focus Group Discussion Interview Guide

1. What does ‘good’ or ‘ideal’ mental health mean to you?

2. How do you know that someone has or may be experiencing a mental illness?

3. How are people with mental illness treated in your community?

4. In your opinion, what are some of the best ways to deal with mental illness?

5. In your opinion, what some ways to encourage people to talk about or seek help for mental illness?

6. In your opinion, what are the things that stop people from speaking about or seeking help for mental illness?

Key Informant Interview Guide

1. I understand that you are a leader in the Arabic-speaking/Mandarin-speaking/Congolese*community. Can you tell me what role you play in your community?
2. The World Health Organisation defines ‘*mental illness’ as a combination of changes in thoughts, perceptions, emotions, behaviour and relationships with others which are typically associated with distress and/or problems functioning in social, work or family activities*. What is your understanding of mental illness and how it applies to members of your community?
3. How are people with mental illness treated in your community?
4. Who do you think Arabic-speaking/Mandarin-speaking/Congolese* people speak to or seek help from for a mental illness?
5. In your opinion as a community leader, what are some of the best ways to deal with mental illness for members of your Arabic-speaking /Mandarin-speaking/Congolese*’ community?
6. In your experience, what are the things that stop Arabic-speaking/Mandarin-speaking/Congolese*’ people from speaking about mental illness or seeking help for mental illness?
7. Thinking about what you said, what are some ways we could encourage more people to speak about and or seek help for mental illness in the Arabic-speaking/Mandarin-speaking/Congolese*’ community?
8. What would make it easier for your community to be supported in mental health?
